# Community based interventions for the prevention and control of Non-Helmintic NTD

**DOI:** 10.1186/2049-9957-3-24

**Published:** 2014-07-31

**Authors:** Jai K Das, Rehana A Salam, Ahmed Arshad, Hasina Maredia, Zulfiqar A Bhutta

**Affiliations:** 1 Division of Women & Child Health, The Aga Khan University, Karachi, Pakistan; 2 Brown University, Providence, RI, USA; 3 Center of Excellence in Women & Child Health, The Aga Khan University, Karachi, Pakistan; 4 Center for Global Child Health, Hospital for Sick Children, Toronto, Canada

**Keywords:** NTDs, Non-helminthic, Community based interventions

## Abstract

In this paper, we aim to systematically analyze the effectiveness of community based interventions (CBI) for the prevention and control of non-helminthic diseases including dengue, trypanosomiasis, chagas, leishmaniasis, buruli ulcer, leprosy and trachoma. We systematically reviewed literature published up to May 2013 and included 62 studies in this review.

Findings from our review suggest that CBI including insecticide spraying; insecticide treated bednets and curtains; community education and cleanliness campaigns; chemoprophylaxis through mass drug administration; and treatment have the potential to reduce the incidence and burden of non-helminthic diseases. Lack of data limited the subgroup analysis for integrated and non-integrated delivery strategies however, qualitative synthesis suggest that integrated delivery is more effective when compared to vertical interventions; however, such integration was possible only because of the existing vertical vector control programs.

Community delivered interventions have the potential to achieve wider coverage and sustained community acceptance. Eradicating these diseases will require a multipronged approach including drug administration, health education, vector control and clean water and sanitation facilities. This would require high level governmental commitment along with strong partnerships among major stakeholders.

## Introduction

As discussed in paper 1 of this series [1], non-helminthic infections are a group of viral (dengue fever), protozoal (African trypanosomiasis, chagas and leishmaniasis) and bacterial (buruli ulcer, leprosy and trachoma) diseases endemic amongst the poorest population in the tropical and sub-tropical regions. These infections can lead to burdensome health consequences accountable for severe economic costs including blindness due to trachoma and disfigurement from leishmaniasis, leprosy and buruli ulcer. Some of these neglected tropical diseases (NTD) like African trypanosomiasis, chagas and dengue fever can even become fatal at the later stages of the disease [2]. For a more thorough discussion on the epidemiology and burden of each of these diseases, please refer to Paper 1 of this series [1].

The World Health Organization (WHO) recommends widespread vector control and environmental management to prevent the spread of vector borne diseases including dengue, trypanosomiasis, chagas, leishmaniasis and trachoma. These should be coupled with mass and selective chemotherapy, community participation, active diseases surveillance, health education, capacity building and training of community health workers (CHW), provision of drugs, surgical treatment and rehabilitation for deformities [3]. For trachoma, WHO recommends SAFE strategy for prevention and management of trachoma, which includes lid surgery (S), antibiotics (A), facial cleanliness (F), and environmental improvement (E). In this paper, we aim to systematically analyze the effectiveness of community based interventions (CBI) for the prevention and control of non-helminthic NTD including dengue, trypanosomiasis, chagas, leishmaniasis, buruli ulcer, leprosy and trachoma.

## Methods

We systematically reviewed literature published up to May 2013 to identify studies on the effectiveness of CBI for the outlined non-helminthic diseases. Our priority was to select existing randomized, quasi-randomized and before/after studies, in which the intervention was delivered within community settings and the reported outcomes were relevant to the diseases under review. A separate search strategy was developed for each disease using appropriate key words, medical subject heading (MeSH) and free text terms. Search was conducted in the PubMed, Cochrane Libraries, Embase, and WHO Regional Databases. Studies that met the inclusion criteria were selected and double data abstracted on a standardized abstraction sheet. Quality assessment of the included randomized controlled trials (RCT) was done according to the Cochrane risk of bias assessment tool [4]. The outcomes of interest for each of the above diseases are outlined in Table 1. We conducted a meta-analysis for individual studies using the software Review Manager 5.1. Pooled statistics were reported as the relative risk (RR) for categorical variables and standard mean difference (SMD) for continuous variables between the experimental and control groups with 95% confidence intervals (CI). We also attempted to qualitatively synthesize the findings reported in the included studies for other pragmatic parameters identified in our conceptual framework including intervention coverage, challenges/barriers, enabling factors, aspects related to integrated delivery, monitoring and evaluations and equity. The detailed methodology is described in paper 2 of the series [5].

**Table 1:** Outcomes analyzed

**Diseases**	**Outcomes analyzed**
Chagas	Peri-domiciliary Infestation Rate
	Domiciliary Infestation Rate
	Chagas serology Rate
Dengue	Dengue Positive Serostatus
	House Index
	Mean Bretreau Index
	Ovitrap Index
Trachoma	Active Trachoma in All Age Groups
	Active Trachoma in Children
	Chlamydia Trachomatis Infection in All Age Groups
	Chlamydia Trachomatis Infection in Children
Leishmaniasis	Incidence of Cutaneous Leishmaniasis
	Incidence of Visceral Leishmaniasis
	Cure Rate for Cutaneous Leishmaniasis
Leprosy	Incidence of Leprosy
	Leprosy Detection Rate

## Review

**Figure 1:**
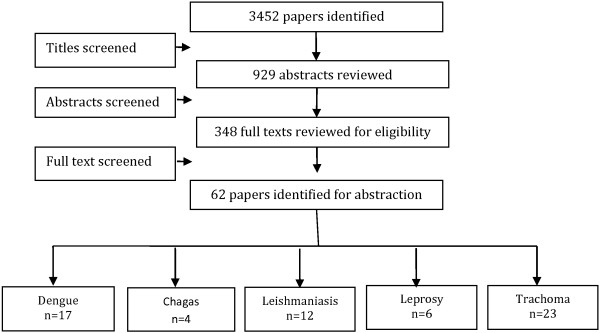
Search flow diagram.

We identified 3452 titles from search conducted in all databases. After screening titles and abstracts, 348 full texts were reviewed, of which 62 studies; 21 RCT and 41 before after studies, were included in the review (Figure 1). The characteristics of the included studies are summarized in Table 2. Of these 62 studies, 17 studies were on dengue, 4 on chagas, 12 on leishmaniasis, 6 on leprosy and 23 on trachoma prevention and control. We did not find any quantifiable data from studies on trypanosomiasis and buruli ulcer to be included in the review. For the 21 RCT included in this review; randomization was adequate in all 21 studies, allocation was concealed in 7, adequate sequence generation was done in 10 while studies provided insufficient information on selective reporting which limited us from making any judgment (Table 3).

**Table 2:** Characteristics of included studies

**Study**	**Study design**	**Country**	**Intervention**	**Target population**	**Integrated/Non-Integrated**
**Dengue**					
Gurtler 2009	Pre-post	Argentina	Preventivelarvicides and insecticide spraying	General population	Non-integrated
Seng 2008	Pre-post	Cambodia	Preventive guppies reared in household water tanks	General population	Non-integrated
Bang 1972	Pre-post	Thailand	Preventive spraying	General population	Non-integrated
Kittayapong 2008	Pre-post	Thailand	Preventive vector control	General population	Non-integrated
Madarieta 1999	Pre-post	Philippines	Preventive Permethrin treated curtains	General population	Non-integrated
Nathan 1982	Pre-post	West Indies	Preventive insecticide spraying	General population	Non-integrated
Neng 1987	Pre-post	China	Preventive growing of Chinese cat fish to consume larvae	General population	Non-integrated
Pant 1971	Pre-post	Bangkok	Preventive Malathion aerosols	General population	Non-integrated
Pai 2006	Pre-post	Taiwan	Preventive cleanliness campaign	General population	Non-integrated
Nam 1997	Pre-post	Vietnam	Preventive community education and cleanliness campaign	General population	Non-integrated
Umniyati 2000	Pre-post	Indonesia	Preventive cleanliness campaign	General population	Non-integrated
Uribe 1984	Pre-post	Columbia	Preventive aerosol applications of Malathion	General population	Non-integrated
Winch 2002	Pre-post	Puerto Rico	Preventive community education program through televised public service announcements and posters	General population	Non-integrated
Kroeger 2006	RCT	Mexico & Venezuela	Preventive insecticide treated curtains	General population	Non-integrated
Vanlerberghe 2009	RCT	Cuba	Preventive insecticide treatments of household items	General population	Non-integrated
Espinoza-Gomez 2002	RCT	Mexico	Preventive spraying and educational campaign	General population	Non-integrated
Lenhart 2008	RCT	Haiti	Preventive insecticide treated bed nets	General population	Non-integrated
**Chagas**					
Arias 1999	Pre-post	Paraguay	Preventivespraying, housing improvement, and a combination of spraying plus housing improvement	General population	Non-integrated
Ferro 1995	Pre-post	Paraguay	Preventive insecticide spraying with lambdacyhalothrin	General population	Non-integrated
Gurtler 2007	Pre-post	Argentina	Preventive community wide spraying with unspecified insecticide	General population	Non-integrated
Gurtler 2004	RCT	Argentina	Preventiveinsecticide spraying	General population	Non-integrated
**Leishmaniasis**					
Alten 2002	Pre-post	Turkey	Preventive Deltamethrin impregnated bed nets	General population	Non-integrated
Dietze 1997	Pre-post	Brazil	Affected dogs were eliminated	General population	Non-integrated
Jalouk 2007	Pre-post	Syria	Preventive ITNs vs. non-treated bed nets	General population	Non-integrated
Yaghoobi-Ershadi 2006	Pre-post	Iran	ITNs, curtains and health education	General population	Non-integrated
Mohebali 2010	Pre-post	Iran	Surveillance followed by treatment of detected cases	Children <12 years	PHC
Safi 2012	Pre-post	Afghanistan	Thermotherapy for Cutaneous Leishmaniasis	General population	Non-integrated
Velasco-Casrejon 1997	Pre-post	Mexico	Therapeutic localized current radio frequency ablation	General population	Non-integrated
Emami 2009	RCT	Iran	ITNs	General population	Non-integrated
Gavgani 2002	RCT	Iran	Community wide application of dog collars	Children	Non-integrated
Picado 2010	RCT	India and Nepal	ITNs	General population	Non-integrated
Reyburn 2000	RCT	Afghanistan	ITNs and Treated chaddars	General population	Non-integrated
Rojas 2006	RCT	Columbia	Deltamethrin bed nets and health education	General population	Non-integrated
**Leprosy**					
Namadi 2002	Pre-post	Nigeria	Integration of services for leprosy detection and elimination through multi-drug therapy	General population	General health systems
Bakker 2005	Pre-post	Indonesia	Preventive Rifampicin chemoprophylaxis	General population	Non-integrated
Rahim 2004	Pre-post	Yemen	Leprosy control program through field searches for cases, clinics, referral centers	General population	Non-integrated
Schuring 2009	Pre-post	Bangladesh	Chemoprophylaxis with Rifampicin and BCG	General population	Non-integrated
Cunha 2008	RCT	Brazil	BCG revaccination of schoolchildren	7-14 years old children	Non-integrated
Moet 2008	RCT	Bangladesh	Rifampicin chemoprophylaxis for close contacts of cases	General population	Non-integrated
**Trachoma**					
Hagan 2009	Pre-post	Ghana	Treatment according to SAFE strategy with Azithromycin	General population	Non-integrated
Alemayehu 2007	Pre-post	Ethiopia	Mass preventive treatment with Azithromycin	General population >1 years	Non-integrated
Astle 2006	Pre-post	Zambia	Treatment of Trachoma through SAFE strategy	General population	Non-integrated
Atik 2006	Pre-post	Vietnam	Treatment through SAFE, SA and S only strategy	Children aged 5–15 years	Non-integrated
Biebesheimer 2009	Pre-post	Eithopia	Preventive annual or biannual mass distribution of azithromycin	General population	Non-integrated
Broman 2006	Pre-post	Tanzania	Preventive mass treatment with azithromycin	General population	Non-integrated
Chidambaram 2006	Pre-post	Ethiopia	Single mass preventive administration of Azithromycin	General population >1 years	Non-integrated
Ewald 2003	Pre-post	Central Australia	Treatment according to SAFE strategy	Children <13 years of age and their households	Non-integrated
Gaynor 2003	Pre-post	Nepal	Single treatment with Azithromycin	Children 1–10 years with their households	Non-integrated
Huguet 2009	Pre-post	Cameroon	Mass preventive administration of Azithromycin drops	General population	Non-integrated
Khandekar 2006	Pre-post	Vietnam	Preventive interventions including improved water andsanitation facilities and increased awareness about active trachoma in the community	General population	Non-integrated
Kumaresan 2003	Pre-post	Multi-country	SAFE strategy	General population	Non-integrated
Lakew 2009	Pre-post	Ethiopia	Mass preventive administration of oral azithromycin	General population	Non-integrated
Schemann 2007	Pre-post	Mali	Mass community-based treatment of all residents, treatment of all children under 11 years of age and of women between 15 and 50 and treatment targeted to inhabitantsof households where at least one child had clinically active trachoma diagnosed with azithromycin	General population	Non-integrated
Edwards 2006	RCT	Ethiopia	Radio messaging, IEC materials, and video van activities along with the SAFE strategy	General population	Non-integrated
Emerson 2004	RCT	Gambia	Preventive intervention group that received regular insecticide spraying or provision of pit latrines (without additional health education) to each household	General population	Non-integrated
Abdou 2010	RCT	Niger	Preventive building of clean water wells and health education	General population	Non-integrated
Fraser-Hurt 2001	RCT	Gambia	Mass administration of Azithromycin vs Topical Tetracycline	General population	Non-integrated
Gebre 2011	RCT	Ethiopia	Preventive mass annual versus twice-yearly azithromycin	General population	Non-integrated
House 2009	RCT	Ethiopia	Preventive mass treatment four times per year vs. treatment delayed until after 1 year vs. routine annual mass administration of azithromycin	children aged between 1 and 10 years	Non-integrated
Melese 2008	RCT	Ethiopia	Biannualvs. annual mass azithromycin administrations	General population	Non-integrated
Schacter 1999	RCT	Egypt, Gambia and Tanzania	Community-wide oral azithromycin treatment or treatment with 1% topicaltetracycline	General population	Non-integrated
West 2007	RCT	Tanzania	Mass treatment with topical tetracycline ointmentplus the face-washing programor treatment only	Children 1–7 years	Non-integrated

**Table 3:** Quality assessment of the included RCTs

**Study**	**Randomization**	**Sequence generation**	**Allocation concealment**	**Blinding of participants**	**Blinding of assessors**	**Selective reporting**
**Chagas**						
Gurtler 2004	Done	Not computerized but done	Not clear	Done	Done	Not clear
**Dengue**						
Kroeger 2006	Done	Not done	Done	Not done	Not clear	Yes
Vanlerberghe 2009	Done	Not computerized but done	Not clear	Not clear	Not clear	Not clear
Espinoza-Gomez 2002	Done	Not computerized but done	Not clear	Not clear	Not clear	No
Lenhart 2008	Done	Not done	Done	Not clear	Not clear	Not clear
**Trachoma**						
Edwards 2006	Done	Not clear	Not clear	Not clear	Not clear	Not clear
Emerson 2004	Done	Not clear	Not clear	Not clear	Done	No
Abdou 2010	Done	Not clear	Not clear	Not clear	Not clear	Not clear
Fraser-Hurt 2001	Done	Not clear	Not clear	Not clear	Not clear	Yes
Gebre 2011	Done	Done	Done	Not clear	Done	Not clear
House 2009	Done	Done	Done	Not clear	Done	Not clear
Melese 2008	Done	Done	Done	Not clear	Done	No
Schacter 1999	Done	Done	Not clear	Not clear	Not clear	Yes
West 2007	Done	Not clear	Not clear	Not done	Done	No
**Leishmaniasis**						
Emami 2009	Done	Done	Not clear	Not clear	Not clear	No
Gavgani 2002	Done	Not done	Not clear	Not clear	Not clear	No
Picado 2010	Done	Not clear	Not clear	Not clear	Not clear	Not clear
Reyburn 2000	Done	Not clear	Not clear	Not clear	Done	No
Rojas 2006	Done	Not clear	Not clear	Not clear	Done	Yes
**Leprosy**						
Cunha 2008	Done	Done	Done	Not done	Not done	Not clear
Moet 2008	Done	Done	Done	Done	Done	No

Included studies mainly focused on community based vector control measures like insecticide spraying and insecticide treated nets (ITN) for dengue, chagas and leishmaniasis; mass drug administration (MDA) for the prevention and treatment of leprosy and trachoma and SAFE strategy for trachoma. Two of the studies focused on removing affected dogs and using insecticide treated dog collars for preventing leishmaniasis [6,7]. All the studies for dengue and chagas targeted general population, while two studies for leishmaniasis [6,8], one for leprosy [9] and five from trachoma [10-14] targeted children less than 15 years of age. Delivery mechanism in most of the studies was non-integrated except for two studies [8,15] in which the intervention was integrated with primary health care (PHC). The primary comparison was between the CBI and routine facility based care or no intervention while, we also attempted to conduct subgroup analysis for the relative effectiveness of preventive and therapeutic drug administration and for the evidences from RCT and pre-post studies, where possible, and reported the results accordingly. Due to limited data we could not conduct an integrated versus non-integrated sub-group analysis. The results are summarized in Table 4.

**Table 4:** Results for overall and sub-group analysis according to type of study and treatment

**Outcomes**	**Estimates (95% CI)**				
	**Combined**	**RCTs**	**Pre-post studies**	**Preventive**	**Therapeutic**
**Chagas**					
Peri-domiciliary Infestation Rate	0.77 [0.53, 1.14]	0.94 [0.67, 1.32]	**0.17 [0.06, 0.48]**	0.77 [0.53, 1.14]	No studies
	8 datasets, 3 studies	4 datasets, 1 study	4 datasets, 2 studies	8 datasets, 3 studies	
Domiciliary Infestation Rate	**0.32 [0.19, 0.55]**	No studies	**0.32 [0.19, 0.55]**	**0.32 [0.19, 0.55]**	No studies
	4 datasets, 2 studies		4 datasets, 2 studies	4 datasets, 2 studies	
Chagas Serology Rate (RR)	**0.78 [0.61, 0.98]**	No studies	**0.78 [0.61, 0.98]**	**0.78 [0.61, 0.98]**	No studies
	4 datasets, 2 studies		4 datasets, 2 studies	4 datasets, 2 studies	
**Dengue**					
House Index	**0.84 [0.81, 0.88]**	No studies	**0.84 [0.81, 0.88]**	**0.84 [0.81, 0.88]**	No studies
	9 datasets, 9 studies		9 datasets, 9 studies	9 datasets, 9 studies	
Ovitrap Index	**0.77 [0.64, 0.92]**	No studies	**0.77 [0.64, 0.92]**	**0.77 [0.64, 0.92]**	No studies
	5 datasets, 3 studies		5 datasets, 3 studies	5 datasets, 3 studies	
Mean Bretreau Index (SMD)	−0.04 [−0.28, 0.19]	−0.04 [−0.28, 0.19]	No studies	−0.04 [−0.28, 0.19]	No studies
	5 datasets, 2 studies	5 datasets, 2 studies		5 datasets, 2 studies	
Dengue Positive Serostatus	**0.31 [0.18, 0.53]**	**0.33 [0.18, 0.60]**	0.14 [0.01, 1.62]	**0.31 [0.18, 0.53]**	No studies
	4 datasets, 4 studies	2 datasets, 2 studies	2 datasets, 2 studies	4 datasets, 4 studies	
**Trachoma**					
Active Trachoma All Age Groups	**0.24 [0.21, 0.26]**	**0.72 [0.59, 0.88]**	**0.15 [0.14, 0.17]**	**0.72 [0.59, 0.88]**	**0.15 [0.14, 0.17]**
	6 datasets, 3 studies	2 datasets, 1 study	4 datasets, 2 studies	2 datasets, 1 study	4 datasets, 2 studies
Active Trachoma in Children	**0.67 [0.64, 0.69]**	**0.86 [0.83, 0.90]**	**0.38 [0.36, 0.40]**	**0.77 [0.74, 0.79]**	**0.32 [0.29, 0.35]**
	20 datasets, 14 studies	6 datasets, 4 studies	14 datasets, 9 studies	13 datasets, 8 studies	7 datasets, 6 studies
Chlamydia Trachomatic infection- All Age Groups	**0.29 [0.27, 0.32]**	**0.28 [0.25, 0.31]**	**0.32 [0.27, 0.37]**	**0.28 [0.25, 0.31]**	**0.36 [0.29, 0.46]**
	10 datasets, 6 studies	5 datasets, 3 studies	5 datasets, 3 studies	7 datasets from 5 studies	3 datasets, 1 studies
Chlamydia Trachomatic infection in Children	**0.21 [0.18, 0.24]**	**0.15 [0.13, 0.19]**	**0.42 [0.31, 0.55]**	**0.21 [0.18, 0.24]**	No studies
	9 datasets, 7 studies	6 datasets, 4 studies	3 datasets, 3 studies	9 datasets, 7 studies	
**Leishmaniasis**					
Incidence Cutaneous Leishmaniasis	**0.42 [0.36, 0.49]**	**0.40 [0.32, 0.51]**	**0.43 [0.35, 0.53]**	**0.42 [0.36, 0.49]**	No studies
	9 datasets, 5 studies	5 datasets, 3 studies	4 datasets, 2 studies	9 datasets, 5 studies	
Incidence of Visceral Leishmaniasis	0.93 [0.83, 1.04]	0.97 [0.84, 1.12]	0.87 [0.73, 1.04]	0.93 [0.83, 1.04]	No studies
	4 datasets, 4 studies	2 datasets, 2 studies	2 datasets, 2 studies	4 datasets, 4 studies	
Cure Rate for Cutaneous Leishmaniasis (RR)	**0.92 [0.88, 0.96]**	No studies	**0.92 [0.88, 0.96]**	No studies	**0.92 [0.88, 0.96]**
	2 datasets, 2 studies		2 datasets, 2 studies		2 datasets, 2 studies
**Leprosy**					
Leprosy Incidence	**0.32 [0.30, 0.34]**	**0.67 [0.49, 0.92]**	**0.31 [0.29, 0.33]**	**0.32 [0.30, 0.34]**	No studies
	8 datasets, 5 studies	1 datasets, 1 studies	7 datasets, 4 studies	8 datasets, 5 studies	
Leprosy detection rate	**1.11 [1.02, 1.21]**	No studies	**1.11 [1.02, 1.21]**	**1.11 [1.02, 1.21]**	No studies
	2 datasets, 2 studies		2 datasets, 2 studies	2 datasets, 2 studies	

Estimates in bold represents statistical significance.

### Quantitative synthesis

**Figure 2:**
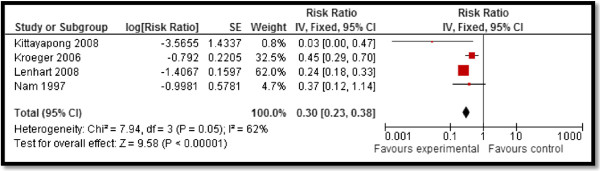
Forest plot for the impact of CBI on dengue seropositive status.

**Figure 3:**
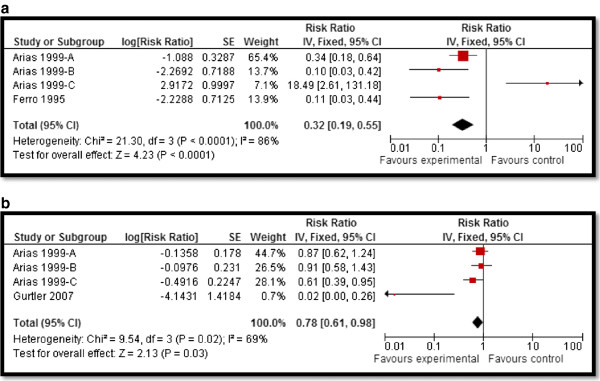
**Forest plot for the impact of CBI on chagas domiciliairy infestation rate and serology. a** and **b.**

**Figure 4:**
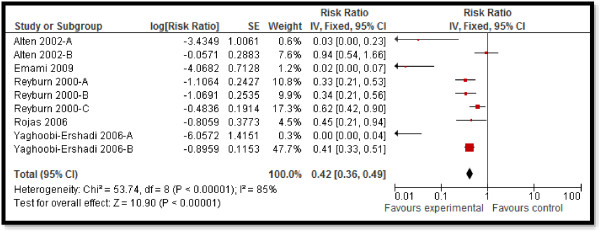
Forest plot for the impact of CBI on incidence of cutaneous leishmaniasis.

**Figure 5:**
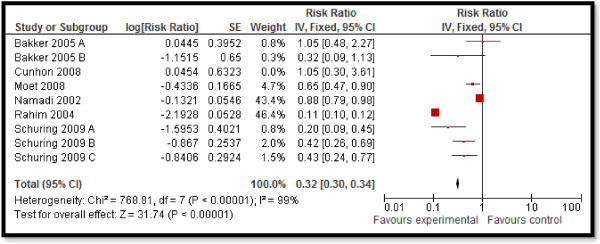
Forest plot for the impact of CBI on incidence of leprosy.

**Figure 6:**
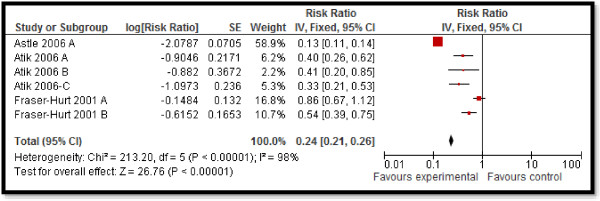
Forest plot for the impact of CBI on active trachoma (all ages).

CBI for dengue preventive measures including use of ITN and curtains significantly reduced dengue positive serostatus by 70% (RR: 0.30, 95% CI: 0.23, 0.38) while community education alone did not have a significant impact (Figure 2). Preventive community based education and cleanliness campaigns reduced ovitrap index by 25% (RR: 0.75, 95% CI: 0.62, 0.91). Insecticide spraying and aerosols significantly reduced house index by 10% (RR: 0.90, 95% CI: 0.86, 0.95) while preventive strategies utilizing guppies in water tank and growth of Chinese cat fish to consume larvae also had significant impact on reducing house index. Bednets and curtains had a non-significant impact however the studies reported spillover effects and non-suitable controls. Community education alone also did not have any impact.For chagas disease, CBI including preventive insecticide spraying with housing improvement (ensure smooth, flat, and crack-free walls and ceiling surfaces and improving openings for ventilation and illumination) had a significant impact with a 68% reduction in domiciliary infestation rate (RR: 0.32, 95% CI: 0.19, 0.55) and a 22% reduction in serology (RR: 0.78, 95% CI: 0.61, 0.98) while it did not show any significant impact on peri-domiciliary infestation rate (Figure 3).For leishmaniasis, CBI including ITN and curtains with education significantly reduced the incidence of cutaneous leishmaniasis by 58% (RR: 0.42, 95% CI: 0.36, 0.49) (Figure 4). Treatment with thermotherapy and radiofrequency resulted in significant 8% (RR: 0.92, 95% CI: 0.88, 0.96) reduction in cure rates of cutaneous leishmaniasis while interventions including surveillance, elimination of dogs, dog collars and ITN had non-significant impact on the incidence of visceral leishmaniasis.For leprosy, treatment with MDA or rifampicin with community education resulted in a 68% reduction in the incidence of leprosy (RR: 0.32, 95% CI: 0.30, 0.34) (Figure 5) and 11% improvement in detection rate (RR: 1.11, 95% CI: 1.02, 1.21). One study evaluating the effect of revaccination of school children with BCG showed no impact on the incidence of leprosy.CBI for trachoma treatment with SAFE strategy and Azithromycin along with community education on hygiene had significant reduction of 76% (RR: 0.24, 95% CI: 0.21, 0.26) (Figure 6) and 33% (RR: 0.67, 95% CI: 0.64, 0.69) in active trachoma among all age groups and children respectively. Chlamydia trachomatic infections also reduced by 71% (RR: 0.29, 95% CI: 0.27, 0.32) and 79% (RR: 0.21, 95% CI: 0.18, 0.24) among all age groups and children respectively. Subgroup analysis for the relative effectiveness of preventive and therapeutic drug administration and for the evidences from RCT and pre-post studies did not show any major differences.

### Qualitative synthesis

Majority of the studies support that community delivered interventions have the potential to achieve wider coverage and sustained community acceptance [16-19] with the combination approach having a more rapid and sustainable effect compared to individual interventions [14,16]. The house-to-house strategy used for the distribution of drugs and commodities also assisted in improving coverage and consequently reducing active disease [14]. Studies also suggest that integrated delivery is more effective when compared to vertical interventions as vertical delivery covers a limited, high-risk population group [16-18]. These integrated programs required strengthened communication and health education components along with broad social participation [17]. However, such integration was reported to be possible only because of the existing vertical vector control programs along with simultaneous strategic development of the infrastructure for improved water and sanitation [17,19].

One of the major reported enabling factor in community directed programs included intersectoral cooperation involving close coordination between external organization, local municipality and the Ministry of Health [10,17]. Another important aspect highlighted in the included studies was the fact that most of the vector control personnel were women from the same community accounting for very low refusals to enter the household premises [17]. To ensure sustainability and preventing future outbreaks, the programs trained a significant number of local human resources along with motivational tools for the continuation of control activities even after the study finished [17,18]. Community involvement, knowledge and education were also highlighted as keys components associated with future sustainability as it encourages the community to continue the use of preventive measures [16,18-20]. It has been reported that conducting an educational campaign is an effective control measure compared to insecticide spraying because in the absence of education, sustainability cannot be ensured. School education has also been found to be an effective strategy [20,21] as school children communicate with their parents about infection prevention measures and increase parental involvement in infection control., More specific messages about the change in behavior and environment need to be directed towards parents [20]. Involvement of children is postulated to promote behavior change in parents, as well as to introduce the children to the concepts of infection prevention at an early age [20]. Mass media and community-wide events should provide appropriate cues to practice specific behaviors on a routine basis and not just during epidemics, while constant positive feedback should be provided to those who are performing the target behaviors [20]. Community delivered programs could turn out to be more cost effective if all vector control tools were locally produced using locally available materials [16]. The low cost and simplicity of impregnated bed nets and curtains ensures their sustainable use in rural communities, given that local people recognize the dangers of vectors, and are amenable to the use of these commodities [22]. Some broader influencing factors included favorable political and sociocultural context that supports discussion of issues affecting health and wellbeing of individuals and community, acquisition of knowledge, and active community involvement in implementation of the program [19].

A few of the barriers reported to hinder effective program implementation and coverage included incomplete surveillance coverage, climatic conditions favorable to the vectors and lack of adequate and sustained community participation [17]. House-to-house larval surveys are typically plagued by difficulties of access, issues of acceptability, coverage and delivery, which frequently compromise the effectiveness of the available vector control tools [17]. For sustainability, surveillance for reintroduction of infectious diseases is necessary to ensure complete eradication [23,24]. There is lack of new, more effective insecticide products that last longer along with the water coverage and storage issues [17]. Certain components of infection control programs require a change in the behaviors of all those at risk as well as the provision of clean water and sanitation. This area has been particularly challenging as change in behavior is slow and provision of water and latrines involves several other sectors and may be costly in resource limiting settings [14]. Maintenance of the hardware and certain health behaviors are also needed to derive health benefits from new housing initiatives [12]. Another important barrier to successful program implementation is the identification of neighborhoods at increased risk of infestation and transmission for developing more cost-effective, targeted control strategies [17]. Effective surveillance coverage of closed or vacant houses also remains to be addressed [18,25].

## Discussion

**Figure 7:**
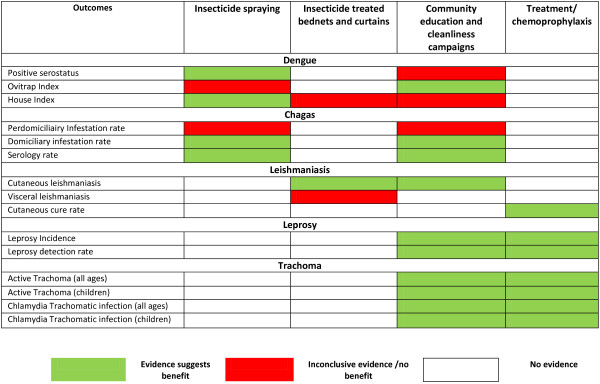
Summary of evidence suggesting areas of benefit according to disease.

Findings from our review suggest that CBI including insecticide spraying; ITN and curtains; community education and cleanliness campaigns; chemoprophylaxis through MDA; and treatment have the potential to reduce the incidence and burden of non-helminthic NTDs. Figure 7 depicts the summary of evidence suggesting areas of benefit by disease. A range of CBI are effective in reducing positive serostatus, house index and ovitrap index for dengue, domiciliary infestation rates and serology for chagas, incidence and cure rates of cutaneous leishmaniasis, incidence and detection of leprosy, active trachoma and chlamydia trachomatic infections. Although some studies did not report significant impacts on Breteau index and peridomiciliary infestation rates but both indices have limitations when used to assess the quantitative impact of control interventions, partly because they are based on presence/absence of immature stages of the larval cycle and it is often difficult to show significant intervention effects on larval indices [17].

Lack of data limited the subgroup analysis for integrated and non-integrated delivery strategies however, qualitative synthesis suggest that community delivered interventions with a combination approach have the potential to achieve wider coverage and sustained community acceptance. It also suggests that integrated delivery is more effective when compared to vertical interventions; however such integration requires pre-existing vertical vector control programs. We did not find any quantifiable data for buruli ulcer and African trypanosomiasis. These two diseases continue to pose great economic burden as the treatment costs for buruli ulcer often exceed per capita government spending on health [26]. Similarly for human African trypanosomiasis, approximately 300,000 cases are reported globally, with approximately 48,000 resulting deaths annually [27].

In January 2012, WHO published a roadmap setting targets for the prevention, control, elimination and eradication of all the NTDs; setting 6 targets for the elimination of 5 NTDs by 2015, and a further 10 targets by 2020, either globally or in selected geographical areas, for 9 NTDs. Since then progress has been made to increase coverage for the MDA. Essential preventive and control measures including community-based early detection, health education and MDA can be achieved through CHW training and capacity building [3]. These programs have been successful in increasing coverage by reaching larger populations without access to healthcare. An example is the control of African trypanosomiasis through active community screening coupled with passive screening at health-care facilities for infections. Much has been done since 2010, however still only 37% of the population in need is being provided with the desired treatment annually while 399 million school age children still in need of treatment [28,29]. This calls for increased scale up of the mass drug campaigns utilizing community platforms to increase coverage. Although CBI are effective in reducing disease burden and improving coverage, there is a major gap in evidence for the effectiveness of integrated community delivered interventions. The major challenges faced include conflict, population growth, vector control, resistance to pesticides and medicines, lack of scale up capacity, lack of research and climate change.

## Conclusion

Eradicating NTDs will require a multipronged approach and our review findings suggest that a range of CBI including drug administration, health education, cleanliness campaigns, vector control and clean water and sanitation facilities have the potential to prevent and control this set of diseases. This would require efforts to overcome the barriers to sustainable implementation including improved surveillance, access and coverage. High level governmental commitment along with strong partnerships among major stakeholders with continuous support by the WHO, United Nations Children’s Fund, World Food Programme and the World Bank, relevant national and international non-governmental organizations and key donors to mobilize resources. A major component of CBI should always be the community itself as success of existing NTD programs depends on community structures, customs, beliefs and values that keep community health worker proud and motivated.

## Abbreviations

CBI: Community based interventions; CHW: Community health workers; HAT: Human African trypanosomiasis; IDoP: Infectious Diseases of Poverty; ITN: Insecticide Treated Nets; MDA: Mass drug administration; MeSH: Medical subject heading; NTD: Neglected tropical diseases; PHC: Primary health care; RCT: Randomized Controlled Trials; WHO: World health organization.

## Competing interests

The authors declare that they have no competing interests.

## Authors’ contributions

ZAB was responsible for designing and coordinating the review. AA and HM were responsible for: data collection, screening the search results, screening retrieved papers against inclusion criteria, appraising quality of papers and abstracting data. RAS and JKD were responsible for data interpretation and writing the review. ZAB critically reviewed and modified the manuscript. All authors read and approved the final manuscript.
